# Spreading of components of mood in adolescent social networks

**DOI:** 10.1098/rsos.170336

**Published:** 2017-09-20

**Authors:** Robert W. Eyre, Thomas House, Edward M. Hill, Frances E. Griffiths

**Affiliations:** 1 Centre for Complexity Science, Zeeman Building, University of Warwick, Coventry CV4 7AL, UK; 2 Zeeman Institute: Systems Biology and Infectious Disease Epidemiology Research (SBIDER), University of Warwick, Coventry CV4 7AL, UK; 3 Warwick Medical School, University of Warwick, Coventry CV4 7AL, UK; 4 School of Mathematics, University of Manchester, Oxford Road, Manchester M13 9PL, UK; 5 Centre for Health Policy, University of the Witwatersrand, Johannesburg, South Africa

**Keywords:** social contagion, emotional contagion, depression, mood

## Abstract

Recent research has provided evidence that mood can spread over social networks via social contagion, but that, in seeming contradiction to this, depression does not. Here, we investigate whether there is evidence for the individual components of mood (such as appetite, tiredness and sleep) spreading through US adolescent friendship networks while adjusting for confounding by modelling the transition probabilities of changing mood state over time. We find that having more friends with worse mood is associated with a higher probability of an adolescent worsening in mood and a lower probability of improving, and vice versa for friends with better mood, for the overwhelming majority of mood components. We also show, however, that this effect is not strong enough in the negative direction to lead to a significant increase in depression incidence, helping to resolve the seeming contradictory nature of existing research. Our conclusions, therefore, link in to current policy discussions on the importance of subthreshold levels of depressive symptoms and could help inform interventions against depression in high schools.

## 1. Background

Depression and other associated mood disorders form an increasing burden upon the health of modern society. The World Health Organization estimates that 350 million people are affected by depression throughout the world, leading to morbidity through a reduced ability to work and socialize, as well as mortality due to suicide and other causes [1]. Evidence suggests mood may spread from person to person via a process known as *social contagion*. Previous studies have found social support and befriending to be beneficial to mood disorders in adolescents [2–5], while recent experiments suggest that an individual’s emotional state can be affected by exposure to the emotional expressions of social contacts [6]. Clearly, a greater understanding of how changes in the mood of adolescents are affected by the mood of their friends would be beneficial in informing interventions tackling adolescent depression.

In recent years, evidence has been found to suggest that some behaviour-based illnesses, such as obesity and smoking cessation, can spread from person to person via social contagion [7–15]. However, such work has come under criticism for being unable to distinguish contagion from other possible phenomena that could confound any positive findings of contagion [16–19]. The two simplest confounding phenomena are homophily, where individuals become friends due to sharing the same behaviour, and shared context, where individuals tend towards the same behaviour whether they are friends or not due to some outside influence [16].

Three of the authors of the current work recently developed a model that distinguishes contagion from homophily and shared context. In this approach, we assess statistically whether the probability of an individual changing between binary states over time forms a better fit to the data when risk is stratified by the number of same or opposing state friends the individual has, or when risk is independent of the state of the individuals friends [20]. This showed that while healthy mood spreads, depression does not, although treating a complex set of mood states as either ‘ill’ or ‘not ill’ can be an oversimplification. Doing this in the case of depression ignores all individuals with subthreshold levels of depressive symptoms, despite their public-health importance [21].

Further, individual component symptoms of depression have not to our knowledge been considered when modelling social contagion, despite being much easier to measure and track in certain cases. These may provide an alternative basis for formulating future interventions. In this study, we, therefore, consider the possibility of social influence upon mood by considering arrays of possible mood states. We first generalize our confounding-robust model to non-binary states, than apply it to data from the National Longitudinal Study of Adolescent to Adult Health (Add Health) [22]. We do this for mood as a whole and for the following seven potential depressive symptoms: anhedonia (loss of interest), poor appetite, poor concentration, dysphoria (sadness), helplessness, tiredness and worthlessness. In general, both high and low values of these measures exhibit social contagion. We also introduce a complementary Gaussian process model that helps to demonstrate why these results are consistent with the observation that depression does not spread in social networks. To conclude, we discuss the possible implications of our results to public policy and health.

## 2. Material and methods

### 2.1. Data

We used data from the first two waves of the in-home interview survey of Add Health, which were performed 6–12 months apart. These included records of adolescents’ in-school friends [22]. We defined the mood state of each individual in our study using their Centre for Epidemiological Studies Depression scale (CES-D) score calculated from the set of 18 CES-D questions asked within the survey [23]. This gave a discrete integer mood state for each individual ranging from 0 to 54, where a higher state indicated a worse mood.

To analyse individual depressive symptoms, we split the total CES-D score into composite parts associated with the subsets of questions related to each symptom. The symptoms analysed were anhedonia (loss of interest), poor appetite, poor concentration, dysphoria (sadness), helplessness, tiredness and worthlessness. The range of states depended on the number of questions related to that symptom. Some, such as poor appetite, only ranged between 0 and 3 while others, such as helplessness, ranged between 0 and 15. In these cases a higher state indicated a worse case of that symptom.

To be included in our study sample analysing mood state and the individual depressive symptoms, at both time points the adolescent student had to be from a saturated school (in which all students were given the in-home interview, eliminating selection bias and ensuring as complete a social network as possible), have given complete answers to all the CES-D survey related questions, and have been the least restricted in the number of school friends they were allowed to give (each student was either asked to list up to five male and five female friends, or was limited to only listing one male and one female friend). This gave us a sample size of 2194 individuals.

### 2.2. Contagion model

If we let a component of mood for an individual at time *t* with *k* friends with better mood and *k*′ friends with worse mood be represented by an integer random variable *X*(*t*), we can imagine a very general probabilistic model for mood in which

2.1 $$\mathrm{Pr}(X(t+1)=x^{\prime }\;|\;X(t)=x)=f(x^{\prime },x,k,k^{\prime }).$$

In practice, finding an appropriate function *f* for such a general model becomes too difficult and so we will normally need to consider special cases of this general model. In our earlier work [20], we considered only binary states *X*(*t*)=*D* for an individual with depressive symptoms at time *t* and *X*(*t*)=*N* for a non-depressed (healthy) individual, and sought to distinguish between sigmoidal dependence on the number of friends in a given state and no such dependence.

Such an approach is robust to confounding from homophily and shared context due to the stratification of the state transition probability over time by the number of contagious state friends, but it does not account for the possibility of different numerical scores for the CES-D components. To relax this assumption we now let *X*(*t*) be an integer, and consider a trinomial model specified by three probabilities: the probability of increasing state, the probability of decreasing state and the probability of remaining in the same state.

2.2 $$\begin{array}{rl} \mathrm{Pr}(X_{i}(t+1)>X_{i}(t)) & =p,\\ \mathrm{Pr}(X_{i}(t+1)<X_{i}(t)) & =q\\ \;\text{and}\;\mathrm{Pr}(X_{i}(t+1)=X_{i}(t)) & =1-p-q. \end{array}\}$$

We examined whether these probabilities were dependent on the states of an individual’s friends relative to their own at the first time point by comparing two different functional forms for *p* and *q*. The first was conditioned on the number of friends of an individual who had better/worse mood at the first time point, *k*. This took the form of a discrete S-shaped (sigmoidal) function, appropriate for behavioural contagion being a type of complex contagion [15,24,25], with the following mathematical formulation:

2.3 pk=α+β∑l=0k(10l)γl(1−γ)1−landqk=δ+ϵ∑l=0k(10l)ζl(1−ζ)1−l.}

The second functional form for *p* and *q* was independent of the states of the friends:

2.4 $$p_{k}=\alpha \;\mathrm{and}\;q_{k}=\delta .$$

Using each possible combination of these two functional forms gave us four models to compare. Model 1, where *p*_*k*_ and *q*_*k*_ are given by (2.3), has both increasing and decreasing state being dependent on friend states. Model 2, where *p*_*k*_ and *q*_*k*_ are given by (2.4), has neither increasing nor decreasing state being dependent on friend states. Model 3, where *p*_*k*_ is given by (2.3) and *q*_*k*_ by (2.4), has increasing state alone being dependent on friend states. Model 4, where *p*_*k*_ is given by (2.4) and *q*_*k*_ by (2.3), has decreasing state alone being dependent on friend states. These models, with separate model variants conditioned either on higher scoring friends or lower scoring friends, were each fitted to the Add Health data using maximum-likelihood estimation (MLE) with likelihood functions of the form

2.5 $$L(\alpha ,\delta ,\ldots )=\prod_{i}\mathrm{Pr}(X_{i}(t+1)|\{X_{j}(t)\}),$$

(the precise form in terms of *p*_*k*_ and *q*_*k*_ can be found in the electronic supplementary material). Competing models were compared using their Akaike information criterion (AIC) values in order to find the preferred model in each case [26]. Sensitivity of these results when applying different thresholds for which the mood of two individuals (or the same individual at two different time points) was considered equal were then analysed, i.e. at what values must *Δ*_*i*_=|*X*_*i*_(*t*+1)−*X*_*i*_(*t*)| and *Δ*_*ij*_=|*X*_*i*_(*t*)−*X*_*j*_(*t*)| be greater than to indicate that individual *i* has changed mood and individual *i* has different mood from individual *j*, respectively.

Goodness-of-fit tests were performed by comparing observed residuals of state changes to the empirical distributions of residuals found using parametric bootstrapping on the fitted model. Details of this can be found in the electronic supplementary material.

### 2.3. Gaussian process model

The model described above deals with the probability of a change of state X(t)→X(t+1) given a number *k* of better or worse scoring friends. We might instead assume that the initial state *X*(*t*) and the state at the next time point *X*(*t*+1) are known and treat the number of friends *k* (either better or worse) as the random variable to be modelled. As we will see, the data in this form is very noisy and so we smooth the function *k*(*X*(*t*),*X*(*t*+1)) using Gaussian process regression [27]. This semi-parametric statistical method allows patterns in the data to become manifest without imposing too much *a priori* structure on the model. More detail is given in the electronic supplementary material.

## 3. Results and discussion

**Figure 1.**
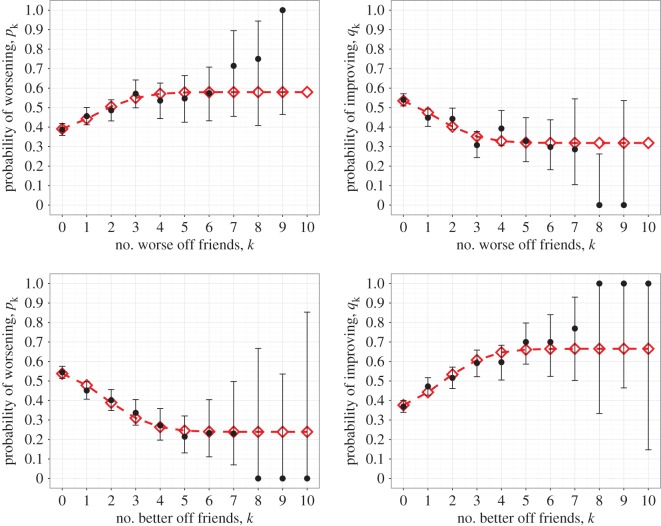
Probability of changing mood state as a function of either the number of better mood (lower state) friends or the number of worse mood (higher state) friends. Observed data (black circles) are shown with 95% CIs alongside the results of fitting (red diamonds) the state change model to the Add Health data. Four possible models, with increasing and decreasing state each being either dependent or independent on the number of higher or lower state friends, were fitted to the data. The preferred model in this case for both better mood and worse mood friends had both increasing and decreasing state being dependent (parameter values provided in table 1 and AIC values in electronic supplementary material, Table S1).

In the case of overall mood (i.e. the total CES-D score) for both conditioning on higher state and on lower state friends the preferred model turns out to be Model 1 where both increasing and decreasing state are dependent on the friends’ states (figure 1 and table 1). This leads to the conclusion that, for US adolescents, the greater number of worse mood friends they have the more likely they are to get worse in mood and the less likely they are to get better, and vice versa for better mood friends. The fact that mood is influenced socially by both worse and better mood friends in this way gives support to social contagion of mood.

**Table 1.** Fitted parameter values for the preferred model of mood state change, with upper and lower values for their 95% CIs calculated using the asymptotic normality of maximum-likelihood estimates.

	worse mood friends model			better mood friends model		
parameter	value	lower limit	upper limit	value	lower limit	upper limit
*α*	0.3718	0.3227	0.4208	0.5556	0.5146	0.5967
*β*	0.2079	0.1442	0.2715	−0.3166	−0.3801	−0.2531
*γ*	0.2120	0.1048	0.3193	0.2497	0.1785	0.3209
*δ*	0.5579	0.5075	0.6083	0.3545	0.3112	0.3977
*ϵ*	−0.2391	−0.3012	−0.1771	0.3108	0.2460	0.3756
*ζ*	0.2090	0.1176	0.3004	0.2337	0.1608	0.3065

The sensitivity analysis examining the effect of applying different thresholds for which the mood of two individuals (or the same individual at two different time points) is considered equal, *Δ*_*ij*_ and *Δ*_*i*_ respectively, revealed that the values required for *Δ*_*ij*_ and *Δ*_*i*_ to result in Model 1 no longer being selected as the preferred model, and therefore evidence of general mood contagion disappearing from the analysis, was 10. Note that under these conditions the majority of individuals are set as never changing mood and most as being the same mood. This indicates that the finding from the baseline analysis is robust against possible noise in the data, and that support is indeed given to general contagion of mood.

**Figure 2.**
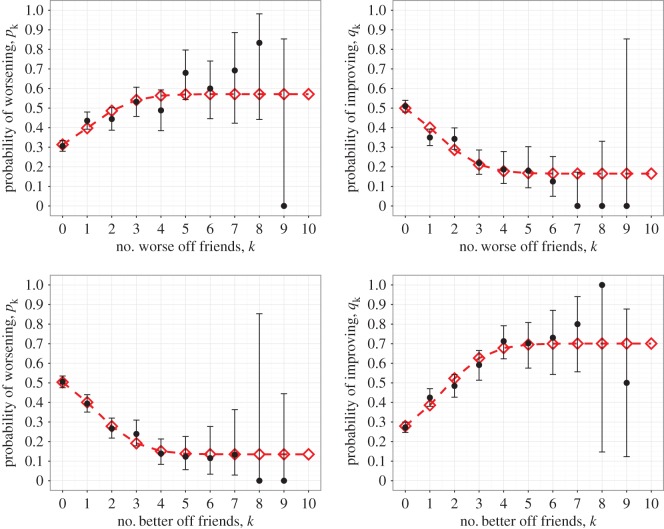
Probability of changing helplessness state as a function of either the number of less helpless (lower state) friends or the number of more helpless (higher state) friends. Observed data (black circles) are shown with 95% CIs alongside the results of fitting (red diamonds) the state change model to the Add Health data. Four possible models, with increasing and decreasing state each being either dependent or independent on the number of higher or lower state friends, were fitted to the data. The preferred model in this case for both less helpless and more helpless friends had both increasing and decreasing state being dependent. Most other depressive symptoms had similar results (parameter values provided in table 2 and AIC values in electronic supplementary material, Table S2).

**Figure 3.**
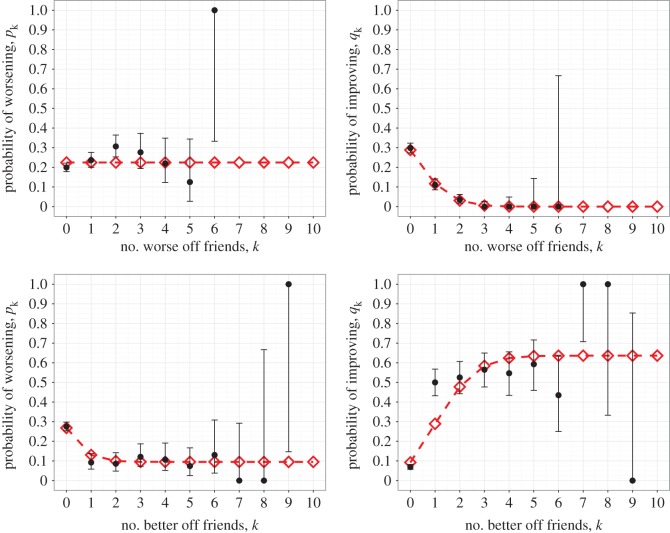
Probability of changing appetite state as a function of either the number of better appetite (lower state) friends or the number of worse appetite (higher state) friends. Observed data (black circles) are shown with 95% CIs alongside the results of fitting (red diamonds) the state change model to the Add Health data. Four possible models, with increasing and decreasing state each being either dependent or independent on the number of higher or lower state friends, were fitted to the data. The preferred model in this case for better appetite friends had both increasing and decreasing state being dependent. For worse appetite friends, it had decreasing state alone being dependent (parameter values provided in table 3 and AIC values in electronic supplementary material, Table S2).

Similar results were found for the individual depressive symptoms of anhedonia, poor concentration, dysphoria, helplessness, tiredness and worthlessness (see figure 2 and table 2 for helplessness, further results in the electronic supplementary material). The only exception is poor appetite (see figure 3 and table 3). In this instance we found that Model 4 was preferred, where decreasing state alone is affected by friend states, although we caution against over-interpretation of this result since AIC is not an infallible method for model selection. In all cases, no models were rejected under the goodness-of-fit tests (details in the electronic supplementary material).

**Table 2.** Fitted parameter values for the preferred model of helplessness state change, with upper and lower values for their 95% CIs calculated using the asymptotic normality of maximum-likelihood estimates.

	worse mood friends model			better mood friends model		
parameter	value	lower limit	upper limit	value	lower limit	upper limit
*α*	0.2772	0.2240	0.3305	0.5423	0.4949	0.5897
*β*	0.2940	0.2296	0.3584	−0.4074	−0.4625	−0.3523
*γ*	0.1889	0.1132	0.2646	0.2096	0.1597	0.2596
*δ*	0.5394	0.4890	0.5898	0.2415	0.1990	0.2841
*ϵ*	−0.3743	−0.4320	−0.3167	0.4591	0.3956	0.5226
*ζ*	0.2009	0.1459	0.2559	0.2215	0.1720	0.2710

**Table 3.** Fitted parameter values for the preferred model of appetite state change, with upper and lower values for their 95% CIs calculated using the asymptotic normality of maximum-likelihood estimates.

	worse mood friends model			better mood friends model		
parameter	value	lower limit	upper limit	value	lower limit	upper limit
*α*	0.2245	0.2070	0.2420	0.5430	-0.0067	1.0928
*β*	—	—	—	-0.4479	-0.9835	0.0877
*γ*	—	—	—	0.0477	-0.0283	0.1236
*δ*	0.4429	0.3637	0.5220	0.0000	-0.0548	0.0548
*ϵ*	-0.4429	-0.5218	-0.3640	0.6362	0.5732	0.6992
*ζ*	0.0999	0.0772	0.1225	0.1754	0.1323	0.2185

Although the results (figures 1–3 and the further results shown in the electronic supplementary material) show a particular shape to the mood change probabilities over the number of worse and better mood friends, due to the large confidence intervals about the data for high numbers of friends (caused by the lack of data in these regions) most conclusions that could be inferred from these shapes would not be particularly robust. Yet they do appear to highlight the absence of thresholds on the number of friends exhibiting a worse or better mood to result in a contagion effect, which is unusual for complex contagion.

**Figure 4.**
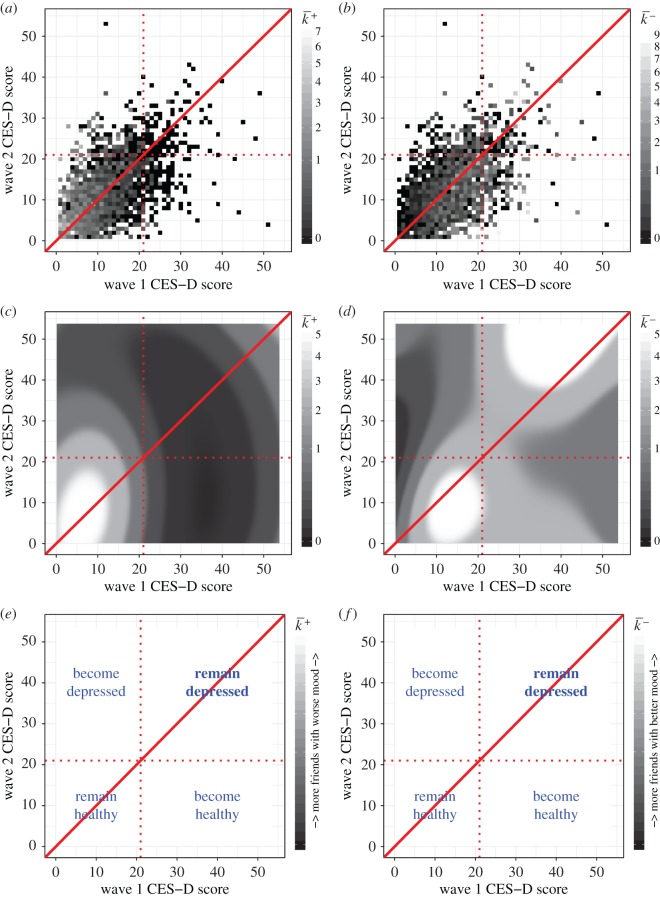
Wave 1 and Wave 2 Centre for Epidemiological Studies depression scale (CES-D) score for (*a*,*b*) our empirical sample and (*c*,*d*) Gaussian process model. Column (*a*,*c*,*e*) is coloured by mean number of friends with worse mood ${\bar{k}}^{+}$ and column (*b*,*d*,*f*) is coloured by mean number of friends with better mood ${\bar{k}}^{-}$. The light regions in these plots show where individuals with greater numbers of worse or better mood friends, and therefore those we expect to experience a stronger contagion effect, are concentrated. The set of states for those who have not changed in state is shown by the diagonal solid red line. The gender-averaged threshold boundary between the states of depressed and not depressed for each wave are shown by the dashed red lines, and the plots (*e*,*f*) show how to interpret the delineated quadrants. We see that individuals with more friends with worse mood (corresponding to higher scoring friends) are contained in the bottom left quadrant, meaning they remain below the depression threshold at both time points with any negative shift in mood caused by contagion seldom enough for the individual to transition to being classified as having depressive symptoms. Individuals with more better mood friends (corresponding to lower scoring friends) are spread out over the bottom two quadrants, meaning that they relatively often improve in mood to such an extent that they cross from being classed as depressed to being healthy in wave 2.

These results superficially contradict our earlier work finding that healthy mood spreads while depression does not [20]. However, our Gaussian process model shows that most of the individuals with a greater number of higher scoring friends who were initially below the threshold for depression remained that way at the second time point, while the individuals with a greater number of lower scoring friends are more spread out in their score combinations such that many that started off above the threshold for depression passed below the threshold at the second time point (figure 4). This suggests that both better and worse moods are contagious, but while better mood is contagious enough to push individuals over the boundary from depressed to not depressed, worse mood is not contagious enough to push individuals into becoming depressed. Consequently, we would not expect to find contagion-like characteristics for depression using a binary model.

We, therefore, observe a difference between depression, which we found not to spread, and relatively low mood below the threshold for depression, which we found did spread. This supports the view that there is more to clinical depression than simply low mood (although the latter may be indicative of the former). It is also in keeping with a tendency for a reduction in the normal social interactions that lead to spreading of mood during an episode of depression [28].

Of existing studies by other authors, the work of Hill *et al.* [7] is closest to ours, and using a different dataset these authors concluded that ‘neutral’ moods did not spread but both ‘content’ (threshold CES-D score 12 on the positively worded questions only) and ‘discontent’ (threshold CES-D score 16) moods did. This work tested models of the form *p*_*k*_=*α*+*βk* using an ordinary least-squares fitting approach, selecting a spreading model if the *p*-value for a slope-free null hypothesis is under 0.05. While we argue that our methodology using a complex contagion of the form (2.3), maximum-likelihood estimation and information-theoretic model selection is preferable to such an approach, we believe that the most important difference with the results presented here is our use of a CES-D threshold score of 20 (or 21) for presumptive depression—and in particular that the spreading of ‘discontent’ at CES-D scores in the 16–20 range is consistent with our results about the spreading of subthreshold levels of depressive symptoms.

The results found here can inform public health policy and the design of interventions against depression in adolescents. Subthreshold levels of depressive symptoms in adolescents is an issue of great current concern as they have been found to be very common, to cause a reduced quality of life and to lead to greater risk of depression later on in life than having no symptoms at all [29–31]. Understanding that these components of mood can spread socially suggests that while the primary target of social interventions should be to increase friendship because of its benefits in reducing of the risk of depression, a secondary aim could be to reduce spreading of negative mood.

Our study comes with certain limitations. As noted above, we were not able to formulate from first principles a fully general model for the components of mood as a function of friends’ moods. We were also unable to learn such a model from data due to the sample size of the study being constrained by the necessity of constructing as complete a friendship network as possible. The friendship network itself may also not be complete. However, as the majority of individuals failed to list the maximum number of friends allowed this implies the network may in fact approach completeness.

We anticipate that future work can further enhance these models in order to cope with a wider range of datasets and more realistically reflect the mechanisms underlying social contagion. Furthermore, we hope that these insights can be used to drive improvements in public health policy and practice.

## Supplementary Material

Supplementary Material

## Data accessibility

The data analysed in this study were obtained on licence from Add Health. Requests for restricted-use data access can be made to Add Health at: http://www.cpc.unc.edu/projects/addhealth/contracts or at health@unc.edu.
