# Supplementary material for: Spreading of components of mood in adolescent social networks
Source: R Soc Open Sci. 2017 Sep 20;4(9):170336. doi: 10.1098/rsos.170336 (PMC5627080; doi:10.1098/rsos.170336)
Supplement: Supplementary Material [file rsos170336supp1.pdf]

Spreading of mood and depressive symptoms over adolescent friendship networks: Supplementary Material

R W Eyre   T House   E M Hill   F E Griffiths

Contents

1 Maximum-likelihood estimation **1**

2 Gaussian process model **1**

3 Further results **1**

4 Goodness-of-fit **6**

4.1 Residual error calculation 6

4.2 Simulations 6

4.3 Results 6

1 Maximum-likelihood estimation

In order to fit the four different models, specified by all the possible combinations of the two different functional forms of p_k (the probability of increasing state given k higher / lower scoring friends) and q_k (the probability of decreasing state given k higher / lower scoring friends) specified in the main article, we performed maximum-likelihood estimation. This was done once for conditioning on higher scoring friends, and once for conditioning on lower scoring friends. The likelihood took the form

$$L\left(\mathbf{x},\mathbf{y}|\mathbf{p},\mathbf{q},\mathbf{N}\right)=\prod_{k}\binom{N_k}{x_k,y_k,N_k-x_k-y_k}p_k^{x_k}q_k^{y_k}\left(1-p_k-q_k\right)^{N_k-x_k-y_k} \tag{1}$$

where x_k was the number of individuals with k higher / lower scoring friends who worsened, y_k was the number of individuals with k higher / lower scoring friends who improved, and N_k was the total number of individuals with k higher / lower scoring friends.

2 Gaussian process model

When modelling the (average) number of friends k dependent on the mood states of individuals at each of the two timesteps $X(t)$ and $X(t+1)$ the data proves very noisy. In order to draw conclusions from this data we smoothed the function $k\left(X(t),X(t+1)\right)$ using Gaussian process regression. This is a semi-parametric Bayesian method that allows us to perform a regression of a set of outputs on to a set of inputs while making minimal assumptions of the shape of this regression relationship. More detail of the method can be found in [1].

In order to take into account the fact that k has both an upper and lower bound, we used warped Gaussian process regression [2]. In this variation of the method we pass the output k through a warping function in order to transform it to an infinite and continuous variable that the regression performs better in learning. The warping function we used was the inverse probit function.

The smoothing was performed using a squared exponential covariance function with lengthscales constrained to have a minimum value of 20 each in order to ensure high levels of smoothing. The hyperparameters of the covariance function were fitted to the data using maximisation of the marginal likelihood.

3 Further results

Alongside the results for mood change, helplessness change, and appetite change detailed in the main article, further results were found for other depressive symptoms. The symptoms in question were anhedonia, poor concentration, dysphoria, tiredness, and worthlessness. Like before, four models were fitted to the data and compared to find the preferred model. Model 1, where both worsening and improving in the given symptom are dependent on the symptom levels of friends. Model 2, where neither worsening or improving are dependent on the symptom levels of friends. Model 3, where worsening alone is dependent on the symptom level of friends. Model 4, where improving alone is dependent on the symptom levels of friends.

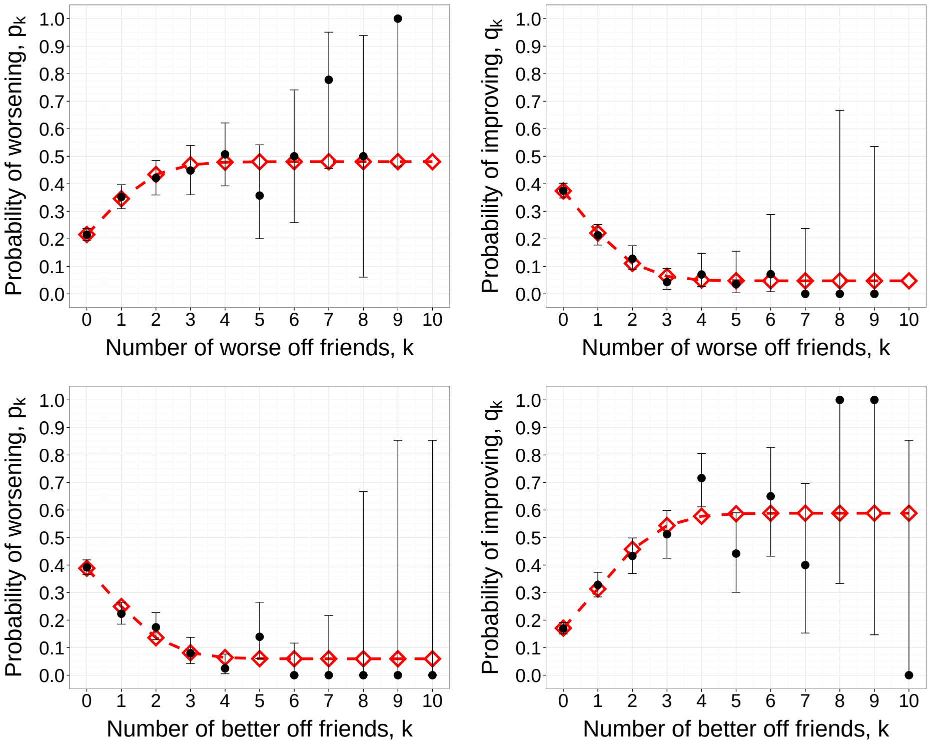

Figure S1: Probability of changing anhedonia state as a function of either the number of better anhedonia (lower state) friends or the number of worse anhedonia (higher state) friends. Observed data (black circles) is shown with 95% confidence intervals alongside the results of fitting (red diamonds) of the state change model to the Add Health data. Four possible models, with increasing and decreasing state each being either dependent or independent on the number of higher or lower state friends, were fitted to the data. The preferred model in this case for both better anhedonia and worse anhedonia friends had both increasing and decreasing state being dependent (parameter values provided in Table S3 and AIC values in Table S2).

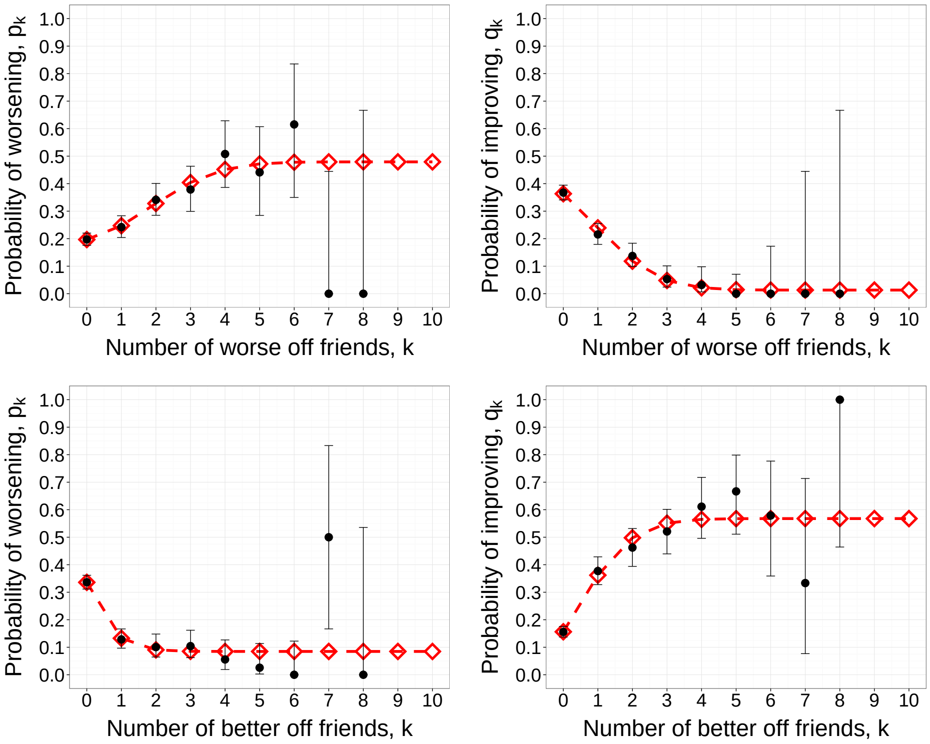

Figure S2: Probability of changing concentration state as a function of either the number of better concentration (lower state) friends or the number of worse concentration (higher state) friends. Observed data (black circles) is shown with 95% confidence intervals alongside the results of fitting (red diamonds) of the state change model to the Add Health data. Four possible models, with increasing and decreasing state each being either dependent or independent on the number of higher or lower state friends, were fitted to the data. The preferred model in this case for both better concentration and worse concentration friends had both increasing and decreasing state being dependent (parameter values provided in Table S4 and AIC values in Table S2).

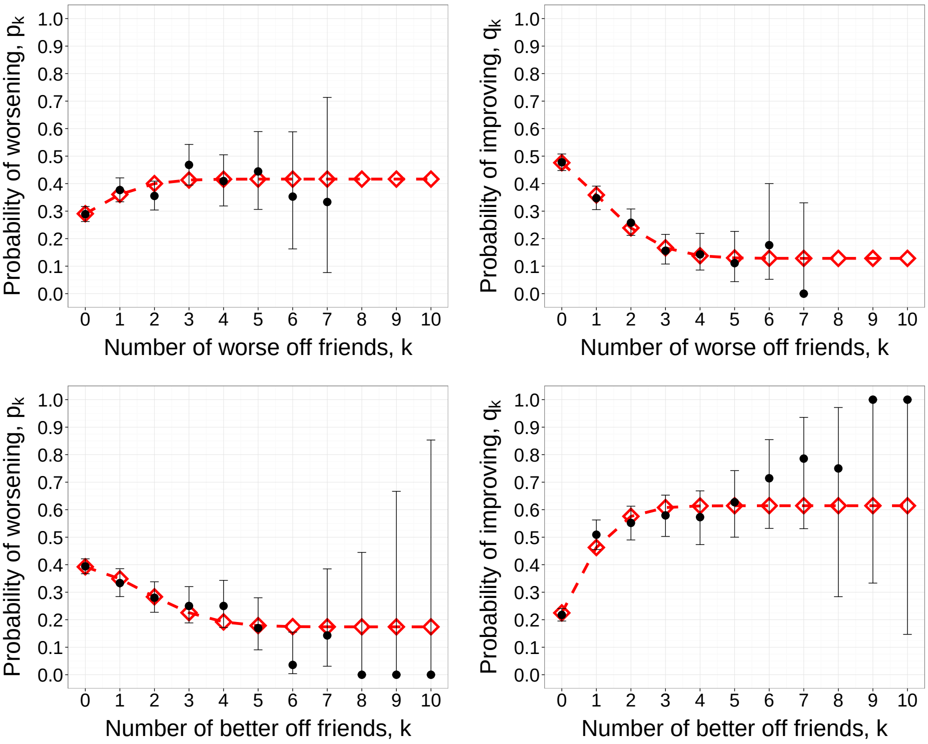

Figure S3: Probability of changing dysphoria state as a function of either the number of better dysphoria (lower state) friends or the number of worse dysphoria (higher state) friends. Observed data (black circles) is shown with 95% confidence intervals alongside the results of fitting (red diamonds) of the state change model to the Add Health data. Four possible models, with increasing and decreasing state each being either dependent or independent on the number of higher or lower state friends, were fitted to the data. The preferred model in this case for both better dysphoria and worse dysphoria friends had both increasing and decreasing state being dependent (parameter values provided in Table S5 and AIC values in Table S2).

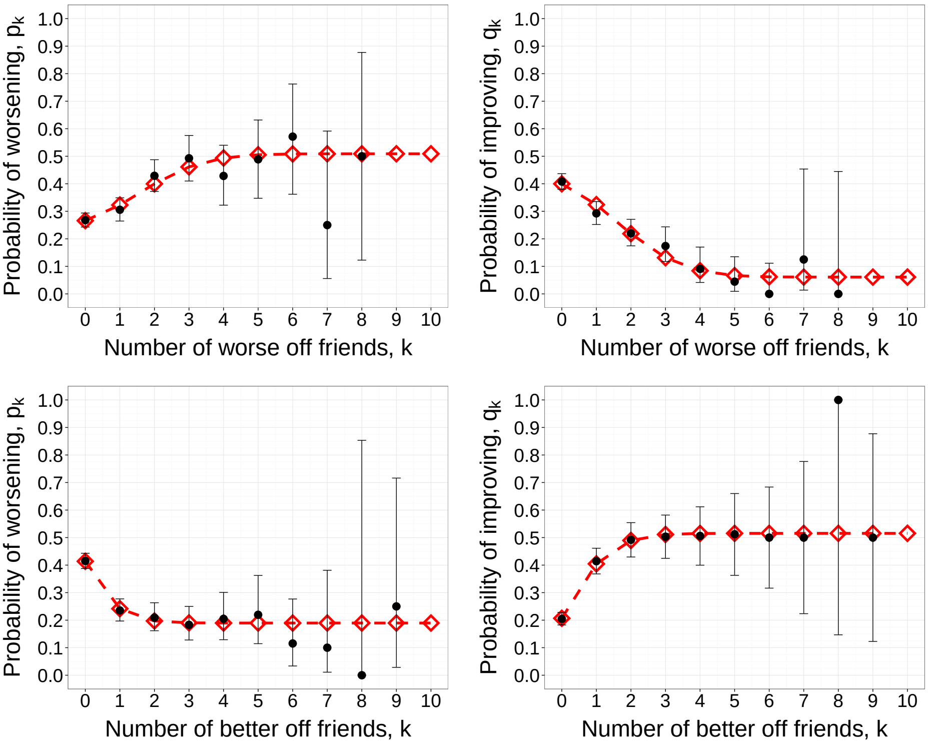

Figure S4: Probability of changing tiredness state as a function of either the number of better tiredness (lower state) friends or the number of worse tiredness (higher state) friends. Observed data (black circles) is shown with 95% confidence intervals alongside the results of fitting (red diamonds) of the state change model to the Add Health data. Four possible models, with increasing and decreasing state each being either dependent or independent on the number of higher or lower state friends, were fitted to the data. The preferred model in this case for both better tiredness and worse tiredness friends had both increasing and decreasing state being dependent (parameter values provided in Table S6 and AIC values in Table S2).

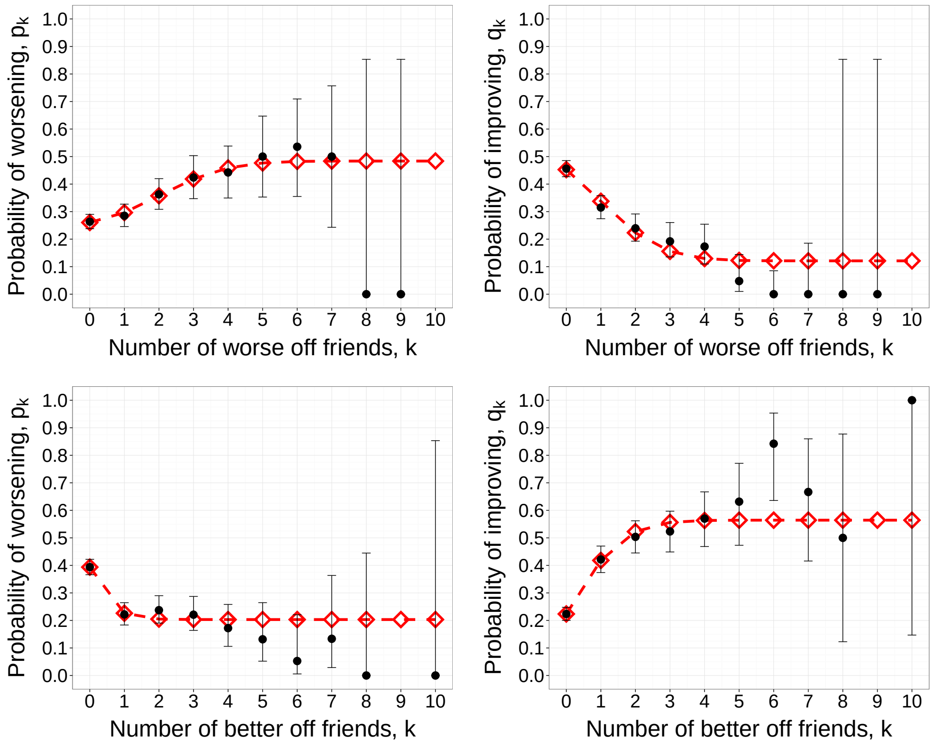

Figure S5: Probability of changing worthlessness state as a function of either the number of better worthlessness (lower state) friends or the number of worse worthlessness (higher state) friends. Observed data (black circles) is shown with 95% confidence intervals alongside the results of fitting (red diamonds) of the state change model to the Add Health data. Four possible models, with increasing and decreasing state each being either dependent or independent on the number of higher or lower state friends, were fitted to the data. The preferred model in this case for both better worthlessness and worse worthlessness friends had both increasing and decreasing state being dependent (parameter values provided in Table S7 and AIC values in Table S2).

Tolerance	Worse mood friends model				Better mood friends model			
	Model 1	Model 2	Model 3	Model 4	Model 1	Model 2	Model 3	Model 4
0	112.6	158.9	162.6	149.1	98.40	188.0	182.2	185.6
2	100.6	186.3	187.0	110.5	103.4	239.0	237.3	157.5
5	77.67	167.8	168.4	86.71	92.42	236.1	220.6	134.3
8	58.41	118.8	117.0	63.64	76.84	204.1	196.0	93.56
10	45.78	78.05	79.80	45.01	68.48	201.1	196.2	77.69

Table S1: Akaiki information criterion (AIC) values for each of the four possible models (as described in the article) for spread of mood dependent on either worse mood or better mood friends, with different tolerance values for the maximum difference between two moods at which those moods are considered equal.

Symptom	Worse mood friends model				Better mood friends model			
	Model 1	Model 2	Model 3	Model 4	Model 1	Model 2	Model 3	Model 4
Anhedonia	94.83	296.4	271.4	191.4	108.8	382.8	320.9	278.8
Appetite	83.19	294.6	298.2	80.18	151.0	658.5	653.7	223.5
Concentration	88.54	284.3	271.4	151.4	99.75	417.9	360.0	260.1
Dysphoria	94.41	246.9	247.3	116.4	108.4	400.0	389.6	162.1
Helplessness	116.7	245.1	245.3	189.9	106.0	312.1	290.8	264.8
Tiredness	103.6	230.8	226.8	156.5	96.26	284.4	266.6	198.3
Worthlessness	107.5	250.6	253.4	148.5	104.4	302.8	297.1	186.4

Table S2: Akaiki information criterion (AIC) values for each of the four possible models (as described in the article) for spread of each component depressive symptom dependent on friends exhibiting either worse or better levels of the symptom.

Parameter	Worse mood friends model			Better mood friends model		
	Value	Lower limit	Upper limit	Value	Lower limit	Upper limit
α	0.1290	0.0398	0.2183	0.4651	0.4016	0.5287
β	0.3512	0.2740	0.4285	-0.4056	-0.4645	-0.3466
γ	0.1307	0.0613	0.2002	0.1534	0.1095	0.1974
δ	0.4696	0.3979	0.5412	0.1077	0.0588	0.1565
ϵ	-0.4222	-0.4872	-0.3572	0.4808	0.4164	0.5453
ζ	0.1381	0.0976	0.1787	0.1834	0.1347	0.2322

Table S3: Fitted parameter values for the preferred model of anhedonia state change, with upper and lower values for their 95% confidence intervals calculated using the asymptotic normality of maximum likelihood estimates.

Parameter	Worse mood friends model			Better mood friends model		
	Value	Lower limit	Upper limit	Value	Lower limit	Upper limit
α	0.1826	0.1514	0.2138	0.7761	0.2525	1.2996
β	0.2967	0.2025	0.3909	-0.6913	-1.2005	-0.1821
γ	0.2621	0.1726	0.3516	0.0442	0.0016	0.0867
δ	0.4205	0.3724	0.4687	0.0164	-0.0796	0.1124
ϵ	-0.4073	-0.4534	-0.3612	0.5514	0.4693	0.6335
ζ	0.1783	0.1420	0.2146	0.1282	0.0810	0.1753

Table S4: Fitted parameter values for the preferred model of concentration state change, with upper and lower values for their 95% confidence intervals calculated using the asymptotic normality of maximum likelihood estimates.

Parameter	Worse mood friends model			Better mood friends model		
	Value	Lower limit	Upper limit	Value	Lower limit	Upper limit
α	0.2345	0.1184	0.3506	0.4055	0.3703	0.4408
β	0.1820	0.0819	0.2822	-0.2313	-0.2896	-0.1730
γ	0.1108	-0.0153	0.2369	0.2491	0.1646	0.3337
δ	0.5278	0.4730	0.5827	0.0000	-0.1494	0.1494
ϵ	-0.3994	-0.4562	-0.3427	0.6145	0.4799	0.7491
ζ	0.1849	0.1350	0.2347	0.0957	0.0560	0.1354

Table S5: Fitted parameter values for the preferred model of dysphoria state change, with upper and lower values for their 95% confidence intervals calculated using the asymptotic normality of maximum likelihood estimates.

Parameter	Worse mood friends model			Better mood friends model		
	Value	Lower limit	Upper limit	Value	Lower limit	Upper limit
α	0.2472	0.2080	0.2864	0.7128	0.2985	1.1271
β	0.2618	0.1864	0.3371	-0.5233	-0.9179	-0.1286
γ	0.2311	0.1390	0.3231	0.0544	-0.0040	0.1129
δ	0.4243	0.3856	0.4630	0.0000	-0.1916	0.1916
ϵ	-0.3633	-0.4132	-0.3134	0.5154	0.3470	0.6837
ζ	0.2367	0.1846	0.2888	0.0873	0.0333	0.1412

Table S6: Fitted parameter values for the preferred model of tiredness state change, with upper and lower values for their 95% confidence intervals calculated using the asymptotic normality of maximum likelihood estimates.

Parameter	Worse mood friends model			Better mood friends model		
	Value	Lower limit	Upper limit	Value	Lower limit	Upper limit
α	0.2509	0.2176	0.2842	0.9864	-0.5547	2.5276
β	0.2328	0.1513	0.3143	-0.7832	-2.3050	0.7386
γ	0.2729	0.1647	0.3812	0.0275	-0.0361	0.0910
δ	0.5046	0.4498	0.5594	0.0608	-0.0703	0.1920
ϵ	-0.3834	-0.4397	-0.3272	0.5035	0.3904	0.6166
ζ	0.1811	0.1308	0.2314	0.1068	0.0565	0.1571

Table S7: Fitted parameter values for the preferred model of worthlessness state change, with upper and lower values for their 95% confidence intervals calculated using the asymptotic normality of maximum likelihood estimates.

The results for these symptoms can be seen in Figures S1 to S5 and tables S3 to S7. The preferred model in all cases for both worse and better friends is model 1, where both worsening and improving are dependent on the friend states. The results are very similar to that of mood as a whole, and lead to the same conclusion that having more friends with a worse case of the given symptom will increase an individuals likelihood of worsening in that symptom and decrease their likelihood of improving, and vice versa for better off friends. Therefore, these symptoms are all generally socially contagious just like mood, and perhaps even spread via the same or similar mechanisms to mood.

The Akaike information criterion (AIC) values used to compare the different possible models for spread of mood and each of the individual symptoms, as well as for the sensitivity analysis of mood difference tolerance (as described in the article), are shown in Tables S1 and S2.

4 Goodness-of-fit

4.1 Residual error calculation

We follow Hill et al. by adapting the Hosmer-Lemeshow test (HL) test, which analyses the distribution of residual errors, to the kind of regression we have performed [3, 4]. The residual error function for our model stratified by number of friends is defined as

$$\mathcal{E} = \left(\sum_{k=0}^{10} \left(Y_k - X_k \left(\theta \right) \right)^2 \right)^{1/2} \tag{2}$$

where Y_k is the number of individuals with k higher/lower state friends who increased/decreased in state in the data, and $X_k(\theta)$ is the modelled number of such individuals given parameters θ. By definition, $\mathcal{E} \geq 0$ and will tend to zero for models that perfectly capture the data.

4.2 Simulations

As an analytical distribution of $\mathcal{E}$ is not available, we use a parametric bootstrap method to simulate the fitted models. This involved applying the models to the initial friendship network from wave 1 of the data $N = 10^4$ times. The proportions of individuals with k higher/lower state friends who increased/decreased in state were extracted and used to calculate residuals.

4.3 Results

Figures S6 to S13 show the observed and simulated residual values for increasing/decreasing state stratified by higher/lower state friends for mood as a whole and the component depressive symptoms for the four different models. Model 1 has both increasing and decreasing state dependent on friend states. Model 2 has neither dependent on friend states. Model 3 has increasing state alone dependent on friend states. Model 4 has decreasing state alone dependent on friend states. Associated p values are given alongside these figures. It should be noted that $\mathcal{E}$ has no asymptotic properties that imply it can be used for model selection in a manor similar to AIC, so no threshold of p value should be considered. Simply, a larger p value shows the model to form a better fit to the data. Overall, these results support our conclusions about which models are preferred in all cases.

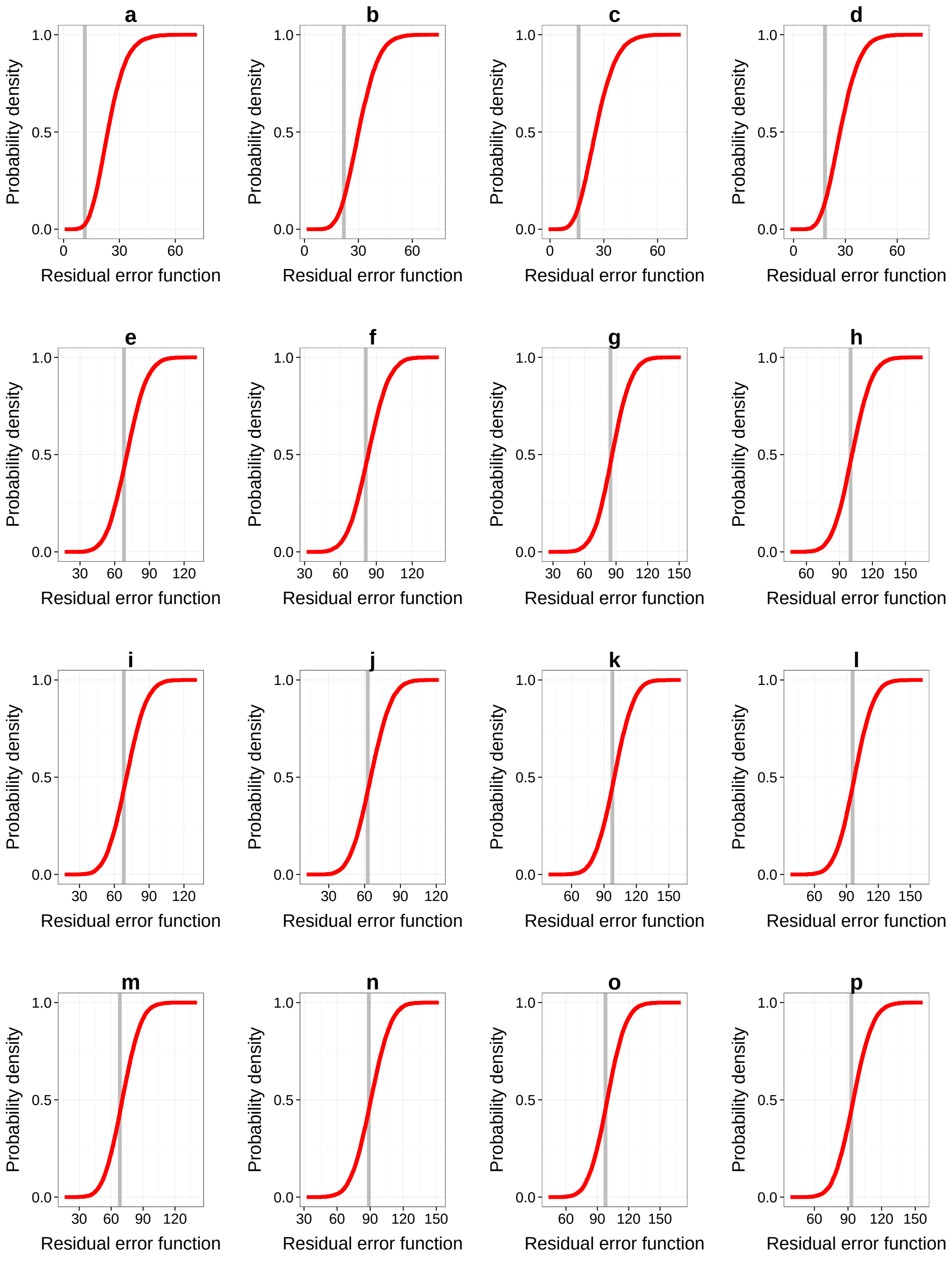

Figure S6: Residual cdfs (in red) with observed residuals (in grey) for the fitted models for total mood. **a** - increasing state residuals for model 1 dependent on higher scoring friends ($p = 0.975$). **b** - decreasing state residuals for model 1 dependent on higher scoring friends ($p = 0.846$). **c** - increasing state residuals for model 1 dependent on lower scoring friends ($p = 0.881$). **d** - decreasing state residuals for model 1 dependent on lower scoring friends ($p = 0.862$). **e** - increasing state residuals for model 3 dependent on higher scoring friends ($p = 0.570$). **f** - decreasing state residuals for model 3 dependent on higher scoring friends ($p = 0.558$). **g** - increasing state residuals for model 3 dependent on lower scoring friends ($p = 0.546$). **h** - decreasing state residuals for model 3 dependent on lower scoring friends ($p = 0.536$). **i** - increasing state residuals for model 4 dependent on higher scoring friends ($p = 0.563$). **j** - decreasing state residuals for model 4 dependent on higher scoring friends ($p = 0.570$). **k** - increasing state residuals for model 4 dependent on lower scoring friends ($p = 0.551$). **l** - decreasing state residuals for model 4 dependent on lower scoring friends ($p = 0.549$). **m** - increasing state residuals for model 2 dependent on higher scoring friends ($p = 0.565$). **n** - decreasing state residuals for model 2 dependent on higher scoring friends ($p = 0.551$). **o** - increasing state residuals for model 2 dependent on lower scoring friends ($p = 0.549$). **p** - decreasing state residuals for model 2 dependent on lower scoring friends ($p = 0.553$).

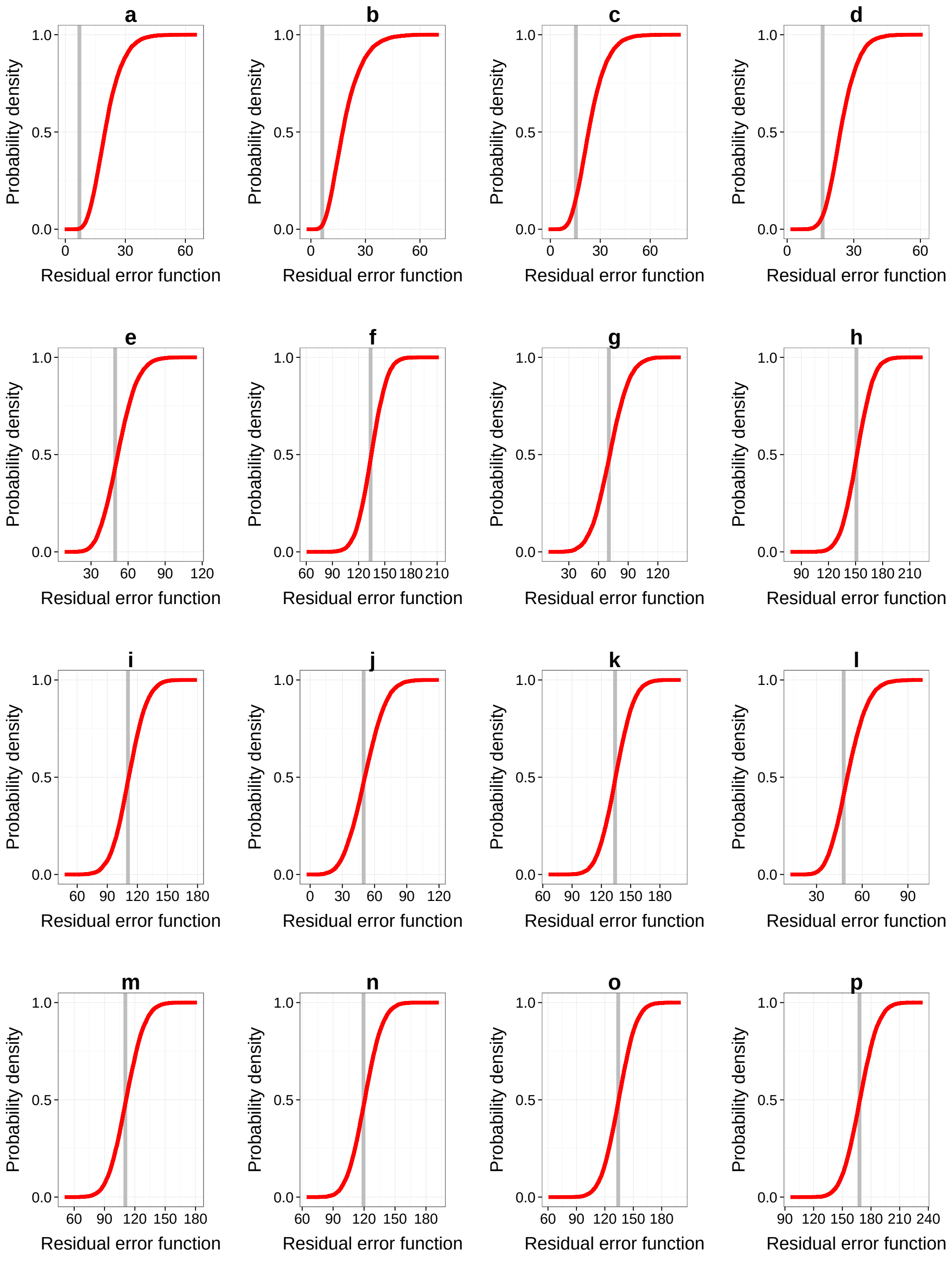

Figure S7: Residual cdfs (in red) with observed residuals (in grey) for the fitted models for anhedonia. **a** - increasing state residuals for model 1 dependent on higher scoring friends ($p = 0.997$). **b** - decreasing state residuals for model 1 dependent on higher scoring friends ($p = 0.980$). **c** - increasing state residuals for model 1 dependent on lower scoring friends ($p = 0.843$). **d** - decreasing state residuals for model 1 dependent on lower scoring friends ($p = 0.932$). **e** - increasing state residuals for model 3 dependent on higher scoring friends ($p = 0.565$). **f** - decreasing state residuals for model 3 dependent on higher scoring friends ($p = 0.524$). **g** - increasing state residuals for model 3 dependent on lower scoring friends ($p = 0.526$). **h** - decreasing state residuals for model 3 dependent on lower scoring friends ($p = 0.516$). **i** - increasing state residuals for model 4 dependent on higher scoring friends ($p = 0.518$). **j** - decreasing state residuals for model 4 dependent on higher scoring friends ($p = 0.526$). **k** - increasing state residuals for model 4 dependent on lower scoring friends ($p = 0.512$). **l** - decreasing state residuals for model 4 dependent on lower scoring friends ($p = 0.590$). **m** - increasing state residuals for model 2 dependent on higher scoring friends ($p = 0.519$). **n** - decreasing state residuals for model 2 dependent on higher scoring friends ($p = 0.534$). **o** - increasing state residuals for model 2 dependent on lower scoring friends ($p = 0.513$). **p** - decreasing state residuals for model 2 dependent on lower scoring friends ($p = 0.509$).

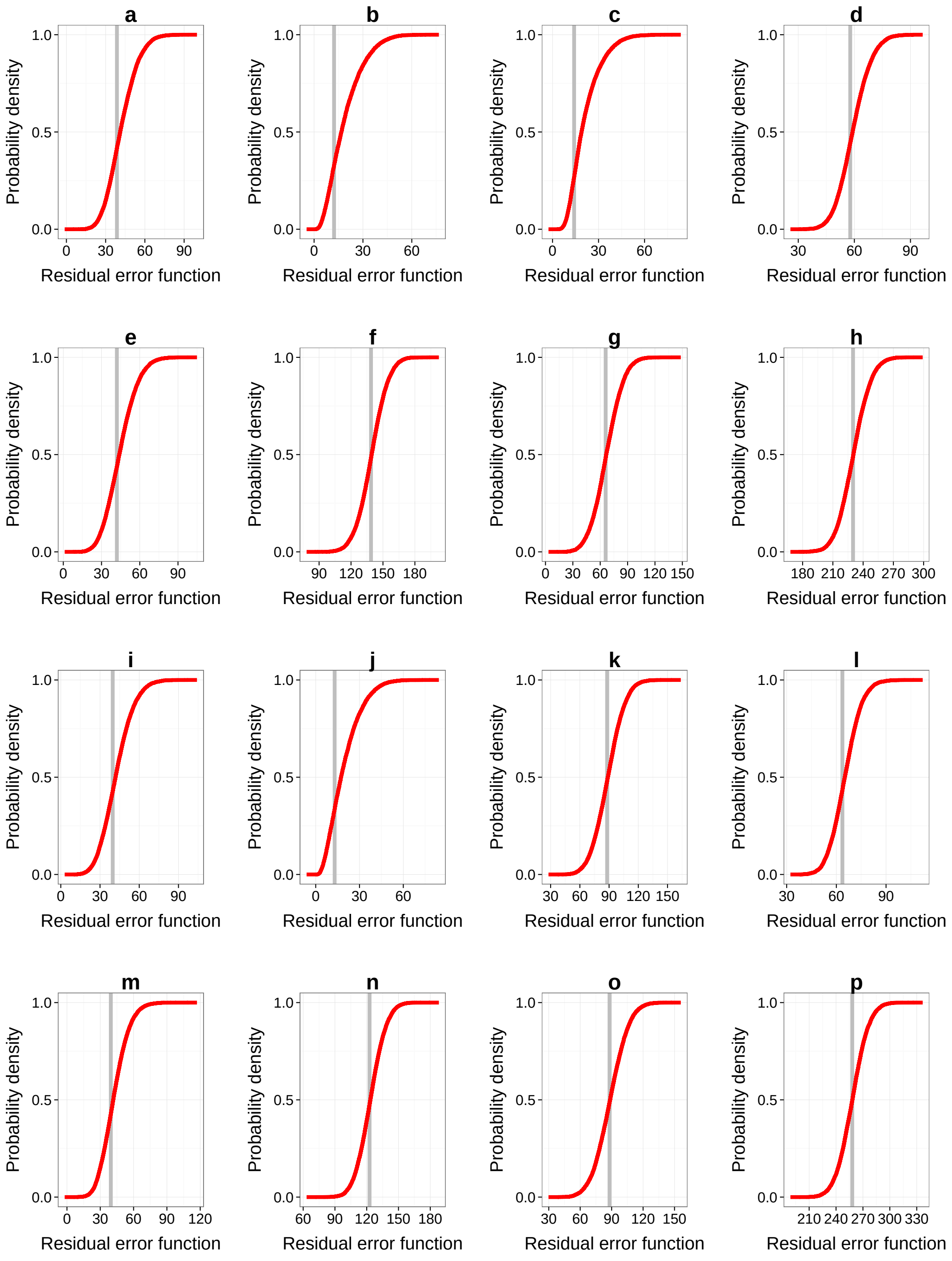

Figure S8: Residual cdfs (in red) with observed residuals (in grey) for the fitted models for appetite. **a** - increasing state residuals for model 1 dependent on higher scoring friends ($p = 0.583$). **b** - decreasing state residuals for model 1 dependent on higher scoring friends ($p = 0.672$). **c** - increasing state residuals for model 1 dependent on lower scoring friends ($p = 0.734$). **d** - decreasing state residuals for model 1 dependent on lower scoring friends ($p = 0.556$). **e** - increasing state residuals for model 3 dependent on higher scoring friends ($p = 0.564$). **f** - decreasing state residuals for model 3 dependent on higher scoring friends ($p = 0.519$). **g** - increasing state residuals for model 3 dependent on lower scoring friends ($p = 0.517$). **h** - decreasing state residuals for model 3 dependent on lower scoring friends ($p = 0.509$). **i** - increasing state residuals for model 4 dependent on higher scoring friends ($p = 0.574$). **j** - decreasing state residuals for model 4 dependent on higher scoring friends ($p = 0.666$). **k** - increasing state residuals for model 4 dependent on lower scoring friends ($p = 0.520$). **l** - decreasing state residuals for model 4 dependent on lower scoring friends ($p = 0.566$). **m** - increasing state residuals for model 2 dependent on higher scoring friends ($p = 0.574$). **n** - decreasing state residuals for model 2 dependent on higher scoring friends ($p = 0.523$). **o** - increasing state residuals for model 2 dependent on lower scoring friends ($p = 0.514$). **p** - decreasing state residuals for model 2 dependent on lower scoring friends ($p = 0.505$).

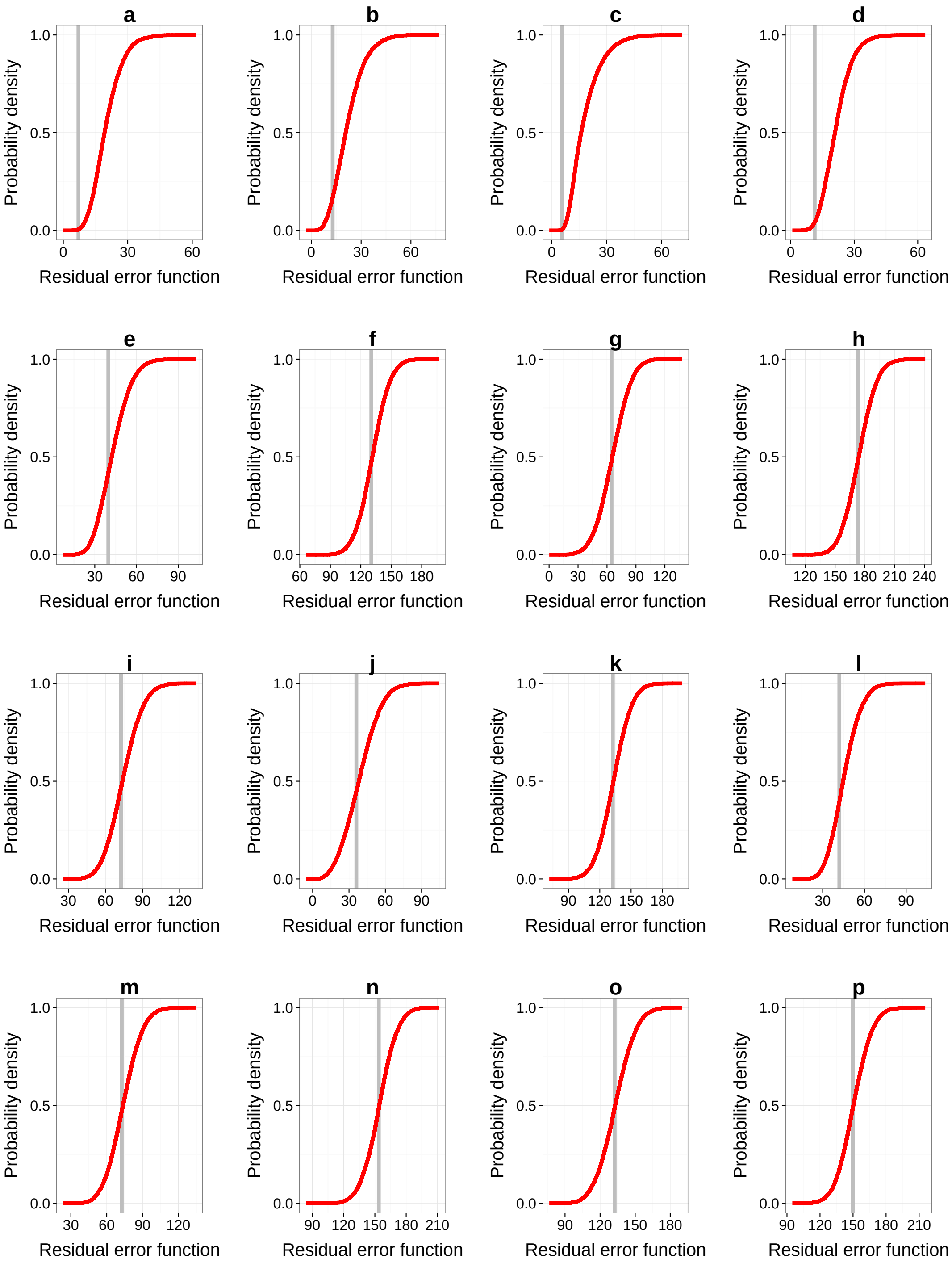

Figure S9: Residual cdfs (in red) with observed residuals (in grey) for the fitted models for concentration. **a** - increasing state residuals for model 1 dependent on higher scoring friends ($p = 0.994$). **b** - decreasing state residuals for model 1 dependent on higher scoring friends ($p = 0.834$). **c** - increasing state residuals for model 1 dependent on lower scoring friends ($p = 0.995$). **d** - decreasing state residuals for model 1 dependent on lower scoring friends ($p = 0.957$). **e** - increasing state residuals for model 3 dependent on higher scoring friends ($p = 0.582$). **f** - decreasing state residuals for model 3 dependent on higher scoring friends ($p = 0.525$). **g** - increasing state residuals for model 3 dependent on lower scoring friends ($p = 0.521$). **h** - decreasing state residuals for model 3 dependent on lower scoring friends ($p = 0.508$). **i** - increasing state residuals for model 4 dependent on higher scoring friends ($p = 0.535$). **j** - decreasing state residuals for model 4 dependent on higher scoring friends ($p = 0.553$). **k** - increasing state residuals for model 4 dependent on lower scoring friends ($p = 0.518$). **l** - decreasing state residuals for model 4 dependent on lower scoring friends ($p = 0.605$). **m** - increasing state residuals for model 2 dependent on higher scoring friends ($p = 0.529$). **n** - decreasing state residuals for model 2 dependent on higher scoring friends ($p = 0.514$). **o** - increasing state residuals for model 2 dependent on lower scoring friends ($p = 0.512$). **p** - decreasing state residuals for model 2 dependent on lower scoring friends ($p = 0.513$).

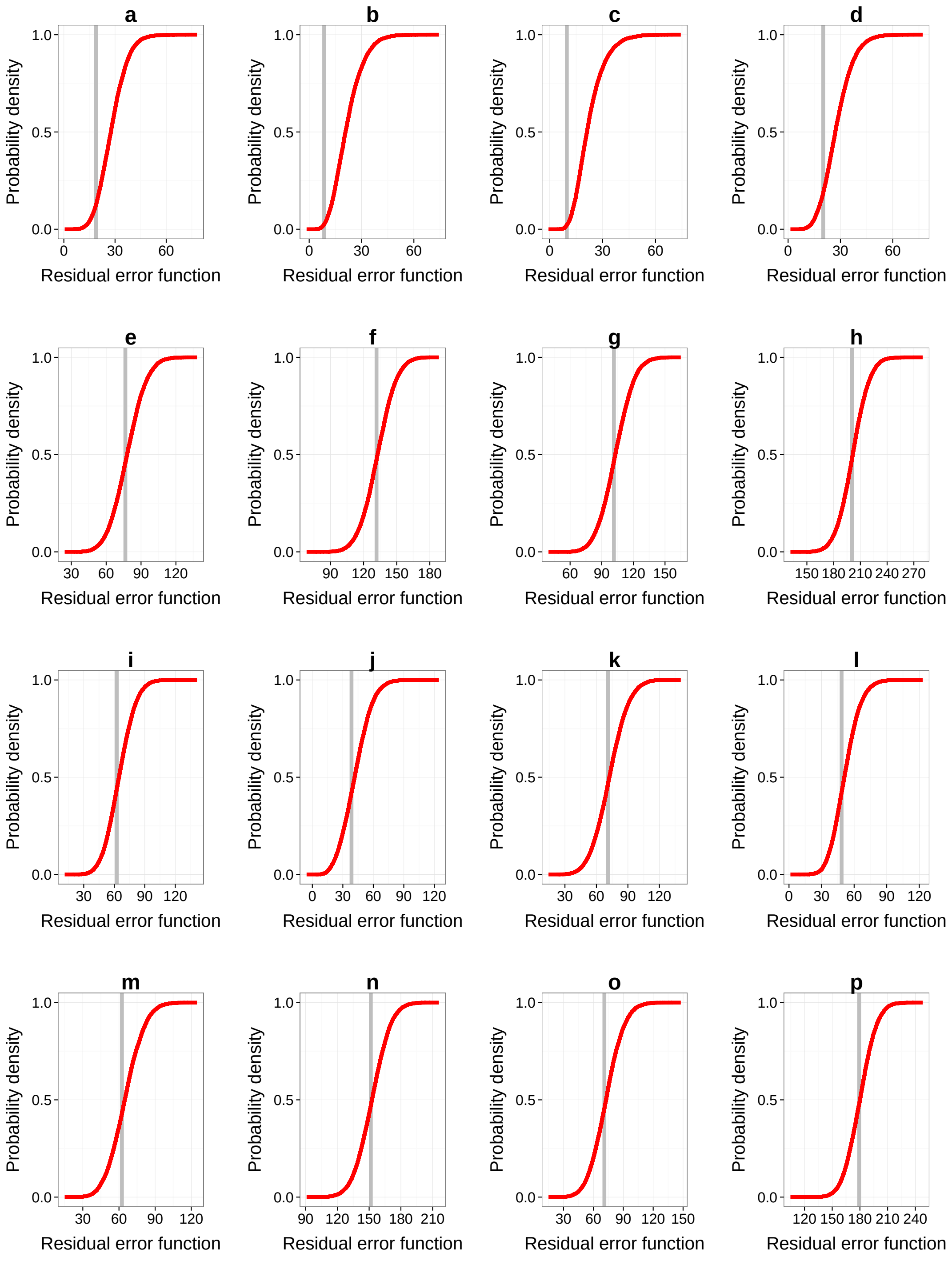

Figure S10: Residual cdfs (in red) with observed residuals (in grey) for the fitted models for dysphoria. **a** - increasing state residuals for model 1 dependent on higher scoring friends ($p = 0.872$). **b** - decreasing state residuals for model 1 dependent on higher scoring friends ($p = 0.974$). **c** - increasing state residuals for model 1 dependent on lower scoring friends ($p = 0.978$). **d** - decreasing state residuals for model 1 dependent on lower scoring friends ($p = 0.812$). **e** - increasing state residuals for model 3 dependent on higher scoring friends ($p = 0.542$). **f** - decreasing state residuals for model 3 dependent on higher scoring friends ($p = 0.527$). **g** - increasing state residuals for model 3 dependent on lower scoring friends ($p = 0.534$). **h** - decreasing state residuals for model 3 dependent on lower scoring friends ($p = 0.521$). **i** - increasing state residuals for model 4 dependent on higher scoring friends ($p = 0.562$). **j** - decreasing state residuals for model 4 dependent on higher scoring friends ($p = 0.579$). **k** - increasing state residuals for model 4 dependent on lower scoring friends ($p = 0.536$). **l** - decreasing state residuals for model 4 dependent on lower scoring friends ($p = 0.579$). **m** - increasing state residuals for model 2 dependent on higher scoring friends ($p = 0.571$). **n** - decreasing state residuals for model 2 dependent on higher scoring friends ($p = 0.531$). **o** - increasing state residuals for model 2 dependent on lower scoring friends ($p = 0.545$). **p** - decreasing state residuals for model 2 dependent on lower scoring friends ($p = 0.522$).

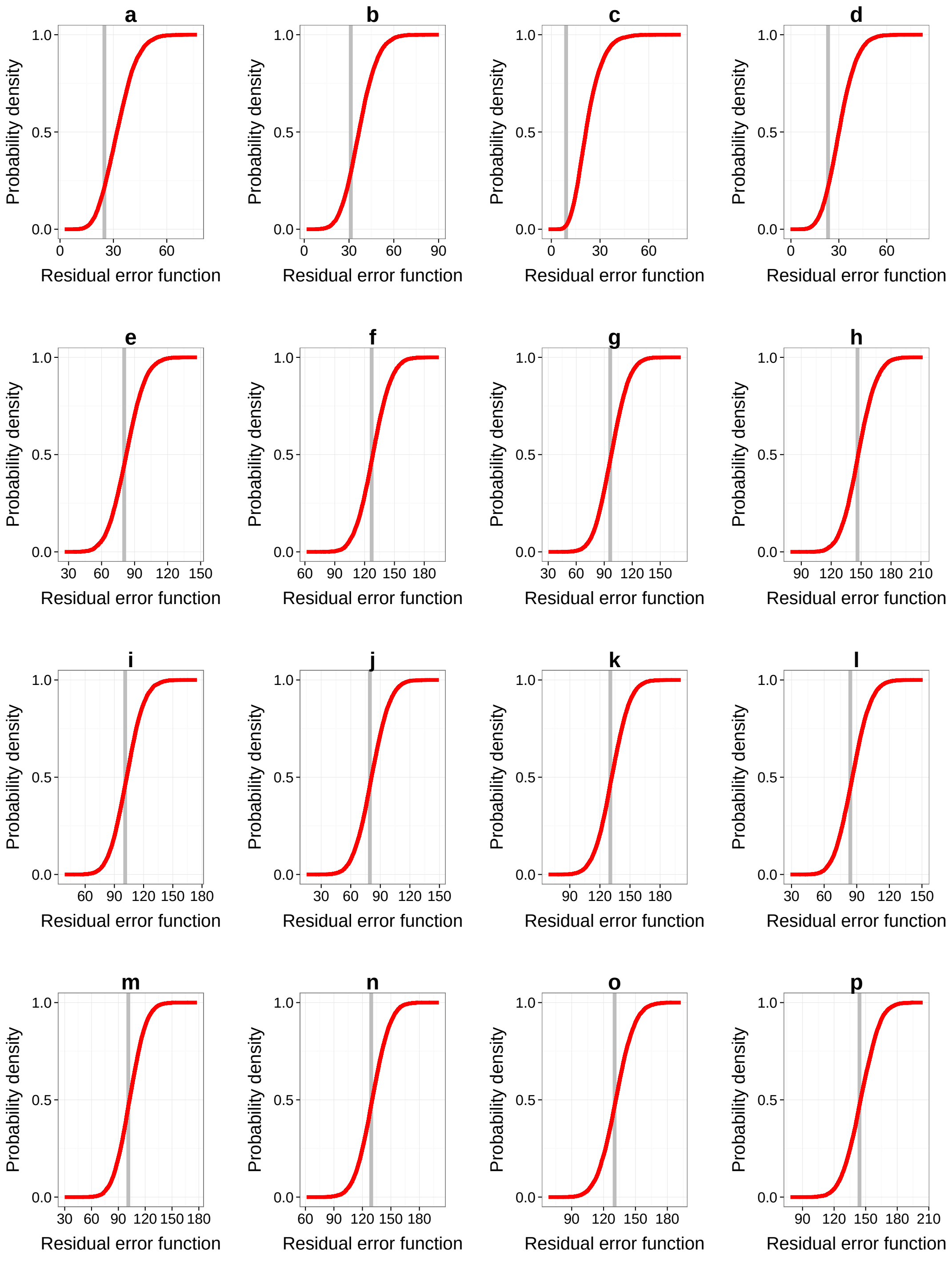

Figure S11: Residual cdfs (in red) with observed residuals (in grey) for the fitted models for helplessness. **a** - increasing state residuals for model 1 dependent on higher scoring friends ($p = 0.786$). **b** - decreasing state residuals for model 1 dependent on higher scoring friends ($p = 0.709$). **c** - increasing state residuals for model 1 dependent on lower scoring friends ($p = 0.981$). **d** - decreasing state residuals for model 1 dependent on lower scoring friends ($p = 0.782$). **e** - increasing state residuals for model 3 dependent on higher scoring friends ($p = 0.553$). **f** - decreasing state residuals for model 3 dependent on higher scoring friends ($p = 0.532$). **g** - increasing state residuals for model 3 dependent on lower scoring friends ($p = 0.528$). **h** - decreasing state residuals for model 3 dependent on lower scoring friends ($p = 0.520$). **i** - increasing state residuals for model 4 dependent on higher scoring friends ($p = 0.540$). **j** - decreasing state residuals for model 4 dependent on higher scoring friends ($p = 0.543$). **k** - increasing state residuals for model 4 dependent on lower scoring friends ($p = 0.534$). **l** - decreasing state residuals for model 4 dependent on lower scoring friends ($p = 0.554$). **m** - increasing state residuals for model 2 dependent on higher scoring friends ($p = 0.534$). **n** - decreasing state residuals for model 2 dependent on higher scoring friends ($p = 0.525$). **o** - increasing state residuals for model 2 dependent on lower scoring friends ($p = 0.531$). **p** - decreasing state residuals for model 2 dependent on lower scoring friends ($p = 0.527$).

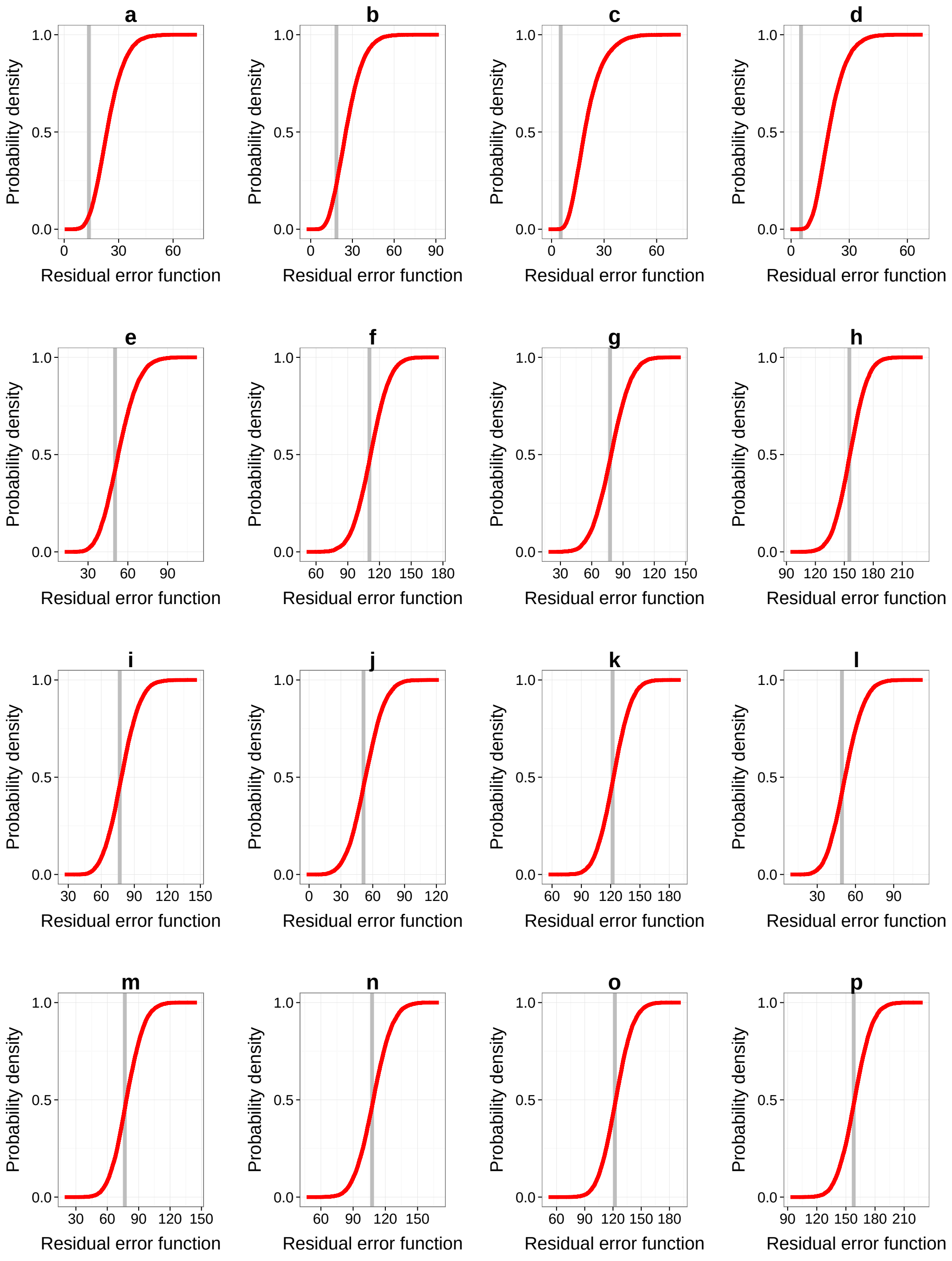

Figure S12: Residual cdfs (in red) with observed residuals (in grey) for the fitted models for tiredness. **a** - increasing state residuals for model 1 dependent on higher scoring friends ($p = 0.931$). **b** - decreasing state residuals for model 1 dependent on higher scoring friends ($p = 0.770$). **c** - increasing state residuals for model 1 dependent on lower scoring friends ($p = 0.998$). **d** - decreasing state residuals for model 1 dependent on lower scoring friends ($p = 0.999$). **e** - increasing state residuals for model 3 dependent on higher scoring friends ($p = 0.581$). **f** - decreasing state residuals for model 3 dependent on higher scoring friends ($p = 0.531$). **g** - increasing state residuals for model 3 dependent on lower scoring friends ($p = 0.528$). **h** - decreasing state residuals for model 3 dependent on lower scoring friends ($p = 0.515$). **i** - increasing state residuals for model 4 dependent on higher scoring friends ($p = 0.543$). **j** - decreasing state residuals for model 4 dependent on higher scoring friends ($p = 0.547$). **k** - increasing state residuals for model 4 dependent on lower scoring friends ($p = 0.524$). **l** - decreasing state residuals for model 4 dependent on lower scoring friends ($p = 0.580$). **m** - increasing state residuals for model 2 dependent on higher scoring friends ($p = 0.544$). **n** - decreasing state residuals for model 2 dependent on higher scoring friends ($p = 0.533$). **o** - increasing state residuals for model 2 dependent on lower scoring friends ($p = 0.523$). **p** - decreasing state residuals for model 2 dependent on lower scoring friends ($p = 0.522$).

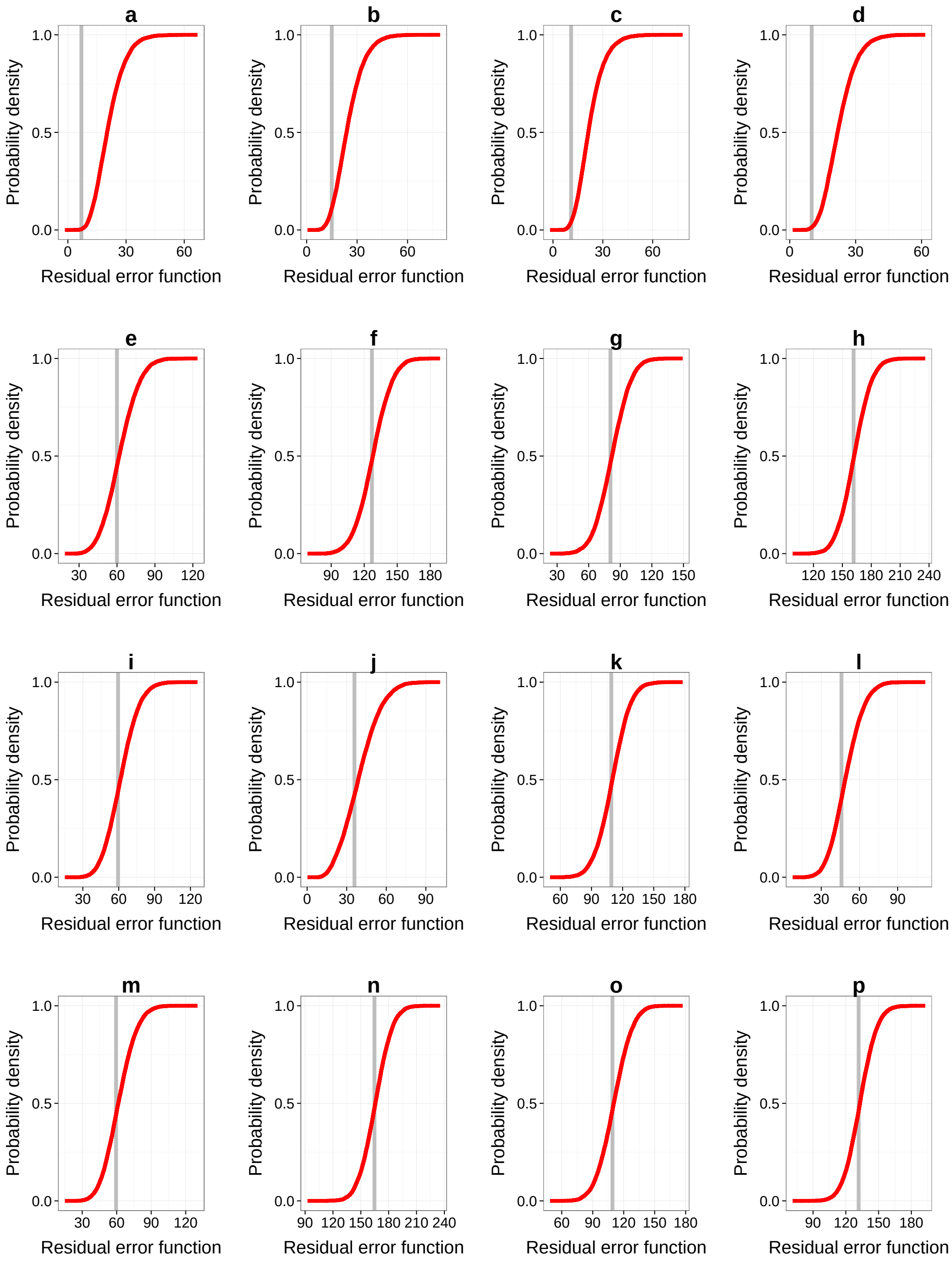

Figure S13: Residual cdfs (in red) with observed residuals (in grey) for the fitted models for worthlessness. **a** - increasing state residuals for model 1 dependent on higher scoring friends ($p = 0.996$). **b** - decreasing state residuals for model 1 dependent on higher scoring friends ($p = 0.887$). **c** - increasing state residuals for model 1 dependent on lower scoring friends ($p = 0.959$). **d** - decreasing state residuals for model 1 dependent on lower scoring friends ($p = 0.987$). **e** - increasing state residuals for model 3 dependent on higher scoring friends ($p = 0.552$). **f** - decreasing state residuals for model 3 dependent on higher scoring friends ($p = 0.525$). **g** - increasing state residuals for model 3 dependent on lower scoring friends ($p = 0.535$). **h** - decreasing state residuals for model 3 dependent on lower scoring friends ($p = 0.514$). **i** - increasing state residuals for model 4 dependent on higher scoring friends ($p = 0.560$). **j** - decreasing state residuals for model 4 dependent on higher scoring friends ($p = 0.579$). **k** - increasing state residuals for model 4 dependent on lower scoring friends ($p = 0.527$). **l** - decreasing state residuals for model 4 dependent on lower scoring friends ($p = 0.598$). **m** - increasing state residuals for model 2 dependent on higher scoring friends ($p = 0.558$). **n** - decreasing state residuals for model 2 dependent on higher scoring friends ($p = 0.526$). **o** - increasing state residuals for model 2 dependent on lower scoring friends ($p = 0.543$). **p** - decreasing state residuals for model 2 dependent on lower scoring friends ($p = 0.538$).

References

[1] Williams, C.K., Rasmussen, C.E.: Gaussian processes for machine learning. MIT Press (2005)

[2] Snelson, E., Rasmussen, C.E., Ghahramani, Z.: Warped gaussian processes. In: NIPS, pp. 337–344 (2003)

[3] Hill, E.M., Griffiths, F.E., House, T.: Spreading of healthy mood in adolescent social networks. Proc R Soc B **282**(1813) (2015). doi:10.1098/rspb.2015.1180

[4] Hosmer, D.W., Lemeshow, S.: Applied logistic regression. John Wiley & Sons, Inc. (2001)
